# Clinical characteristics of adults with alcohol dependence syndrome comorbid with antisocial personality disorder: a cross-sectional study

**DOI:** 10.3389/fpsyt.2024.1397009

**Published:** 2024-09-12

**Authors:** Dominika Jarcuskova, Maria Pallayova, Simona Carnakovic, Maria Frajka, Jan Fidmik, Aneta Bednarova

**Affiliations:** ^1^ 1^st^ Department of Psychiatry, University Hospital of Louis Pasteur and Pavol Jozef Safarik University Faculty of Medicine, Kosice, Slovakia; ^2^ Department of Human Physiology, Pavol Jozef Safarik University Faculty of Medicine, Kosice, Slovakia; ^3^ 2^nd^ Department of Psychiatry, University Hospital of Louis Pasteur and Pavol Jozef Safarik University Faculty of Medicine, Kosice, Slovakia

**Keywords:** depression, anxiety, cognition, antisocial, alcoholism, trauma, sleep

## Abstract

**Introduction:**

Antisocial Personality Disorder (ASPD) is characterized by a pervasive pattern of disregard for and violation of the rights of others, typically emerging by age 15 years and involving behaviors such as deceitfulness, impulsivity, and aggressiveness. The present study sought to examine the prevalence of the comorbid ASPD in adult people with Alcohol Dependence Syndrome (ADS) and identify clinical characteristics associated with ASPD.

**Methods:**

A cross-sectional study of 100 consecutive subjects diagnosed with ADS was conducted. Subjects were examined between August 2023 and September 2023. Various assessments and questionnaires were employed, including the Montreal Cognitive Assessment (MoCA), Alcohol Use Disorders Identification Test (AUDIT), and Structured Clinical Interview for DSM-IV (SCID-II). A computed tomography (CT) scan of the brain was performed on 47.5% participants.

**Results:**

Out of the 100 individuals screened for the study, 20 were excluded. The study found that 35% of the examined study participants had a comorbid ASPD. Individuals with both ADS and ASPD were more likely to be younger, started drinking at an earlier age, had higher hospitalization rates, and scored higher on the AUDIT test (all *P* < 0.05%). Also, they had lower education levels, higher rates of unemployment, and lower marriage rates (all *P* < 0.05%). In addition, they reported more family members with ADS, incarceration, or mental illness and a higher frequency of traumatic experiences (all *P* < 0.05%). Depression, anxiety, stress (all *P* < 0.05%), and sleep problems (*P* = 0.058) were correlated with ASPD. Participants with the comorbid ASPD had lower MoCA scores (*P* = 0.046) and struggled with attention and linguistic subtests compared to subjects with ADS only.

**Conclusion:**

The study highlights the high prevalence of comorbid ASPD in participants with ADS, shedding light on their demographic and psychometric characteristics. Individuals with the comorbid ASPD are more likely to face cognitive deficits, especially in linguistic and attention-related tasks. The findings underline the importance of considering the comorbidity of ASPD in ADS subjects. The study implies that the understanding of the associated risk factors can aid in developing more targeted treatment interventions.

## 1 Introduction

Antisocial Personality Disorder (ASPD) is a pervasive pattern of disregard for and violation of the rights of others, with the average age of onset being 15 years; the diagnosis is based on three or more of the following indicators: failure to conform to social norms concerning lawful behaviors, deceitfulness, impulsivity, irritability, and aggressiveness, reckless disregard for the safety of self or others, consistent irresponsibility, and lack of remorse (1). To meet the diagnosis criteria, an individual must be at least 18 years old and have evidence of a Conduct Disorder (CD) with onset before the age of 15 years (2). The requirement of this childhood criterion serves to portray ASPD as a persistent personality disorder with roots early in development (3). Individuals with ASPD are more prone to be violent; they tend to violate the rights of others, be manipulative, commit crimes, and the disease produces extraordinary societal costs and aggregate social burden (2, 4). The maladaptive behaviors may include suicidal behavior, self-harm, aggression, criminal behavior, and substance misuse (2, 4).

Goldstein et al. (5) also found that individuals with ASPD are more likely to have comorbid substance misuse and other psychiatric disorders. The ASPD is associated with greater odds of any substance use disorder (SUD), specifically alcohol, nicotine, and any drug use disorder (6). Similarly, data from cohorts of prisoners has shown that alcohol dependence syndrome (ADS) shows high comorbidity with ASPD, suggesting that there may be common biological risk mechanisms (7). Furthermore, individuals with ASPD, SUD, and their co-occurrence (ASPD/SUD) have similar personality traits (8). Low agreeableness, low conscientiousness, high impulsivity, high excitement-seeking, low deliberation, and low self-discipline were characteristic of these disorders (8). The ASPD exhibited lower neuroticism than SUD and ASPD/SUD, which is consistent with the high levels of mood and anxiety disturbance often reported in individuals with SUD (8).

Alcohol dependence syndrome is characterized by alcohol use that causes serious impairment. It is characterized by preoccupation with alcohol, impaired control over drinking, usage of alcohol despite adverse consequences, and distortions in thinking. Alcoholism is characterized by craving, loss of control, withdrawal symptoms, and tolerance (9). Alcohol use disorder (AUD) is defined by the Diagnostic and Statistical Manual of Mental Disorders (DSM-5) as “a problematic pattern of alcohol use leading to clinically significant impairment or distress.” An alcohol dependence syndrome aligns symptomatically with the current diagnoses of moderate or severe AUD as per DSM-5. To diagnose a patient with AUD, the patient needs to meet at least two criteria: alcohol usage in larger amounts or for longer periods than intended, unsuccessful efforts to cut down or control alcohol use, spending a long time in obtaining alcohol, using alcohol, or recovering from its effects, failure to fulfil major role obligations due to alcohol, reduction in activities due to alcohol, continued alcohol use despite having persistent or recurrent problems due to alcohol, recurrent alcohol use in situations in which it is physically hazardous, craving, tolerance, and withdrawal. Alcohol Dependence Syndrome is a chronic, relapsing brain disease that presents a significant public health issue (1). There are links between developmental changes in personality and alcohol use (10). Across adolescence and early adulthood, individuals with steeper declines in impulsivity and neuroticism demonstrated steeper declines in problematic alcohol use (10). Individuals with a less substantial decline (or even an increase) in impulsivity and neuroticism had either increases or smaller decreases in problematic alcohol use (10). Similarly, increases in risk-taking behavior across development are associated with increased alcohol use among adolescents (11).

The ADS remains to be the actual topic in the field of psychiatry. Although its exact etiology remains unknown, susceptibility to the disorder is likely multifactorial. The cultural aspects are likely to play a very important role in the prevention and treatment of ADS. In Slovakia, 75% of adults consume alcohol occasionally or regularly. Alcohol is socially more accepted than other substances, and in Slovakia, its consumption is socially promoted (12). Alcoholism is the most commonly seen psychiatric diagnosis amongst Slovakians, and 26.5% of all psychiatric hospitalizations are due to alcohol (13). To our knowledge, the present study is a first study analyzing the prevalence of ASPD amongst the Slovak population. In the present study, we compared the characteristics of subjects with ADS and ADS comorbid with ASPD and compared their cognitive abilities. Knowledge of any cognitive deficits associated with ASPD would be valuable in working toward understanding the neurobiology of this disorder and its relationship with other conditions and developing more targeted treatment interventions.

## 2 Materials and methods

### 2.1 Study design and setting

The present study was a cross-sectional pragmatic study of 100 consecutive adult research participants with a diagnosis of ADS who were hospitalized in the 1^st^ Department of Psychiatry, University Hospital of Louis Pasteur in Kosice. The study was conducted between August 2023 and October 2023. The exclusion criteria were as follows: the diagnosis of intellectual disability, dementia, delirium tremens, substance use disorder (other than alcohol, nicotine, and/or caffeine), the presence of significant withdrawal symptoms (Brief Alcohol Withdrawal score> 3 points), and the subject’s disagreement to participate in the study. Out of the 100 individuals screened for the study, 20 were excluded. The reasons for the exclusion were either meeting the exclusion criteria, not signing the informed consent, or not filling out one or more questionnaires during the examination. The final statistical analysis included only subjects with completed data (80 participants).

The study was reviewed and approved by the Ethics Committee of the University Hospital of Louis Pasteur Kosice (study protocol number: 2023/EK/08045). The participants provided their written informed consent to participate in this study.

### 2.2 Methods

For participants, we obtained sociodemographic and clinical characteristics, including age, sex, and the presence of comorbidities. The information was obtained through the subjects’ clinical interviews and medical records. The study used the following questionnaires: the Brief Alcohol Withdrawal Scale (BAWS), Montreal Cognitive Assessment (MoCA), the Alcohol Use Disorders Identification Test (AUDIT), the Depression, Anxiety, and Stress Scale-42 (DASS-42), the Pittsburgh Sleep Quality Index (PSQI), Structured Clinical Interview for DSM-IV, axis-II (SCID-II), Multifactorial Memory Questionnaire (MMQ), and The Adverse Childhood Experiences - International Questionnaire (ACE-IQ). If patients underwent computed tomography (CT) examination, we obtained these data from their documentation.

#### 2.2.1 Cognition assessment

The MoCA (Montreal Cognitive Assessment) includes tasks focused on attention, visual-constructive abilities, executive functions, immediate and long-term memory, naming, abstract thinking, speech, and orientation (14).

All points for subtasks are added to the total score (14). The maximum number of points is 30 (+ 1 for education) (14). It is also possible to calculate scores for individual cognitive domains, which can be used for differential diagnostic considerations (15). If the resulting score is 26-30, cognitive abilities are normal; if the score is 18-25, there is mild cognitive impairment present; if the participants reach a score of 10-17, we speak of moderate cognitive impairment, and with a score below 10, we diagnose subjects with severe cognitive impairment (14, 15).

#### 2.2.2 Dependence on alcohol assessment

The Alcohol Use Disorders Identification Test (AUDIT) is a 10-item test that asks about domains of alcohol use (16). The first three items refer to risky alcohol consumption, the following three determine the occurrence of possible symptoms of addiction, and the last four statements deal with indicators of harmful alcohol consumption (16). Individual statements are evaluated on a 5-point scale (16). The resulting raw score can be 0–40 points (16). In interpreting the scores obtained, a score of 0–7 indicates low-risk drinking, a score of 8–15 indicates high-risk drinking, a score of 16–19 represents harmful drinking, and a score of 20–40 indicates alcohol dependence (16).

#### 2.2.3 Quality of sleep assessment

We measured sleep quality using the PSQI questionnaire that evaluates sleep quality during the last month (17)⁠. It is a 19-item questionnaire, where each item is assigned a possible score from 0-3, with a higher score indicating a greater problem with sleep (17)⁠. This questionnaire evaluates seven components: Subjective sleep quality, sleep latency, sleep duration, awakening during the night, sleep efficiency, medication use to improve sleep, and daytime dysfunction​​ (17)⁠. Calculating the scores for all seven components gives us the so-called global PSQI score; if it is > 5, it can distinguish individuals with poor sleep from those with good sleep (17)⁠.

#### 2.2.4 Depression, anxiety, stress assessments

In the present study, the DASS-42 was used to measure participant’s affectivity (depression, anxiety, and stress) (18). Participants agree with 42 items on a 4-point Likert scale (18). The depression scale allows us to assess the degree of dysphoria, hopelessness, anhedonia, self-loathing, inactivity, or lack of interest (18). The items in the anxiety scale are mainly focused on physiological reactions but also on experiencing situational anxiety (18). The stress scale contains items measuring the degree of irritability, impatience, excitement, or tension (18). Adding up the points of these items gives us final scores for depression, anxiety, and stress (18). A score of 0-9 points indicates the absence of depression, 10-13 points indicate mild depression, 14-20 points indicate moderate depression, 21-27 points indicate severe depression, and a score above 28 indicates extremely severe depression (18). A score of 0-7 points suggests the absence of anxiety, 8-9 points indicate mild anxiety, 10-14 points indicate moderate anxiety, 15-19 points indicate severe anxiety, and a score above 20 points indicates extremely severe anxiety (18). A score of 0-14 points indicates the absence of stress, 15-18 points indicates mild stress, 19-25 points indicates moderate stress, 26-33 points indicates severe stress, and a score above 34 indicates extremely severe stress (18).

#### 2.2.5 Withdrawal symptoms assessment

The Brief Alcohol Withdrawal Symptoms Scale (BAWS) consists of 5 items (tremor, sweating, agitation, orientation, and hallucinations), with each item rated on a scale from 0 to 3, with higher scores indicating more severe withdrawal symptoms (19).

#### 2.2.6 Metacognition assessment

The Multifactorial Memory Questionnaire (MMQ) was developed to assess several aspects of mnestic functions: emotions associated with memory performance, memory failures, and strategies used in the memory process (20–22). By calculation, we can evaluate subjectively perceived cognition as very low, low, below average, average, above average, good, and very good (21, 22).

#### 2.2.7 Traumatization in childhood assessment

The ACE-IQ (Adverse Childhood Experiences – International Questionnaire) detects traumatic childhood experiences and divides them into 13 categories: physical and emotional abuse, sexual abuse, violence between household members, caring for a family member who is physically or mentally ill, suicidal tendencies, incarceration of a household member, family dysfunction such as the death of one or both parents, divorce or parental separation, emotional and physical neglect, alcohol and drug abuse in the family, collective violence and bullying (23). The resulting score is from 0-13; the higher it is, the more trauma a person has experienced (23).

#### 2.2.8 Antisocial personality disorder assessment

The Structured Clinical Interview for DSM-IV, axis-II (SCID-II, personality disorders) is a diagnostic methodology for ten personality disorders. In our study, we focused only on antisocial personality disorder (24). In a structured interview, we asked the participant 17 questions, some of which were about a social behavior disorder in childhood/adolescence. If the total score was at least 3 points, we asked 14 questions specific to antisocial personality disorder (24). If the subject had at least three symptoms, the diagnosis of antisocial personality disorder was established (24).

### 2.3 Statistical analyses

We analyzed the data using descriptive statistics and cluster analysis. We used the Shapiro-Wilk test to determine the normality of the data distribution. Continuous variables were expressed as medians (p50) and interquartile ranges (IQR = p25 - p75). Categorical variables were expressed as absolute and relative counts. The results of the ordinal logistic regression models are expressed as the probability value (*P*), odds ratio (OR) with 95% confidence interval (95% CI), and standard deviation of the regression coefficient (SD). Patterns between categorical variables were examined using the chi-squared test. A comparison of interval variables between the two groups was performed using the Wilcoxon rank-sum test. A value was considered statistically significant if the *P*-value was less than 0.05. Statistical analyses were performed using Stata Special Edition statistical software Version 13.1 (StataCorp LP, College Station, TX).

## 3 Results

### 3.1 Sample characteristics and univariate analyses

A computed tomography (CT) scan of the brain was performed on 38 (47.5%) participants. Cortical cerebral atrophy was described in 33.8% of participants, and vascular changes were verified in 18.8%. Regarding comorbidities, 31.25% of patients were diagnosed with depressive disorder (25% with the diagnose other depressive episode, 6.25% with Major depressive disorder, recurrent, moderate), 6.25% of patients were diagnosed with Other specific personality disorders, 2.5% of patients were diagnosed with other schizophrenia, 1.25% of patients were diagnosed with Insomnia, not due to a substance or known physiological condition, and 1.25% of patients were diagnosed with Mixed obsessional thoughts and acts. Antisocial personality disorder was found in 35% of participants. More than 46% had no detected cognitive impairment in MoCA, 27.5% had mild cognitive impairment, 21.3% had moderate cognitive impairment, and 5% had severe cognitive impairment. Seventy-five percent of ASPD participants had cognitive impairment in MoCA. Only 8.8% of participants reported subjective problems with cognitive abilities in the MMQ questionnaire. Nearly 65% of participants had the documented pathology in the PSQI questionnaire. More than half of the participants described experiences with emotional abuse (61.3%), the presence of a person addicted to addictive substances in the home environment (57.5%), and observing violence between parents (62.5%). In the DASS-42 Questionnaire, we measured degrees of depression, anxiety, and stress. More than 46% of all participants had no symptoms of depression, 7.5% had only mild symptoms, 10% had moderate symptoms, 10% had severe symptoms, and 26.3% had extremely severe symptoms. Thirty-one point three percent of all participants had no anxiety symptoms, 6.3% had mild symptoms, 16.3% had moderate, 11.3% had severe, and 35% had extremely severe symptoms. More than 46% of all participants had no symptoms of stress, 6.3% had mild, 15% had moderate, 16.3% had severe, and 16.3% had extremely severe symptoms. Depression was reported in 68% of ASPD participants, 64% of participants reported stress, and 79% of participants reported anxiety. Among participants without ASPD, depression was reported in 53.85% of participants, 51.92% of participants reported stress, and 36.54% of participants reported anxiety. Regarding traumatization of ASPD participants, 42.8% reported physical abuse, 75% emotional abuse, 14% contact with sexual abuse, 89% reported alcohol and/or drug abuse in the household, 75% reported witnessing household member treated violently, 54% reported having one or no parent, parental separation or divorce, 29% reported emotional neglect, 43% physical neglect and 32% of ASPD participants reported being bullied. Regarding alcohol withdrawal symptoms, 53.75% of patients had no withdrawal symptoms during the examinations, 12.5% of patients had 1 point, 30% had 2 points, and 3.75% had 3 points on the BAWS scale.

The sociodemographic characteristics of all subjects, with a comparison between the subgroup of subjects with ADS and those with ADS+ASPD, are reported in **Table 1**. The subjects with ASPD were younger, had a lower age of first hospitalization, were hospitalized more often, had a lower education grade, and were employed less frequently (all *P* < 0.05).

**Table 1:** Demographic and clinical characteristics of study participants.

	All (*N*=80)	ADS (*N*=52)	ADS+ASPD (*N*=28)	*P-*value
Males	62 (77.5%)	38 (47.5%)	14 (17.5%)	0.19
Females	18 (22.5%)	24 (30%)	4 (5%)	
Age at first hospitalization, years	42.5 (34-55)	46 (34.5-57.5)	38.5 (30.5-45)	**0.04**
Current age, years	51.5 (38.5-58)	53.5 (39.5-59.5)	45 (36.5-55)	**0.04**
Length of drinking alcohol, years	4 (0.5-8)	3 (0-7)	6 (2-10)	0.127
Number of hospitalizations	4 (2-7)	2.5 (1.5-6)	6 (3.5-12.5)	**0.003**
General education	15 (18.75%)	6 (7.5%)	9 (11.25%)	**0.03**
High school education	55 (68.75%)	37 (46.25%)	18 (22.5%)	
University education	10 (12.5%)	9 (11.25%)	1 (1.25%)	
Unemployed	27 (33.75%)	15 (18.75%)	12 (15%)	**0.047**
Employed	32 (40%)	21 (26.25%)	11 (13.75%)	
Retired pensioners	10 (12.5%)	11 (13.75%)	0 (0%)	
Disability pensioners	11 (13.75%)	5 (6.25%)	5 (6.25%)	
Single	37 (46.25 %)	22 (27.5%)	15 (18.75%)	0.283
Married	25 (31.25 %)	18 (22.5%)	7 (8.25%)	
Widowed	8 (10%)	7 (8.25%)	1 (1.25%)	
Divorced	10 (12.5%)	5 (6.25%)	5 (6.25%)	
Without CT pathology	9 (23.68%)	6 (15.79%)	3 (7.89%)	0.431
Vascular changes in CT	15 (39.47%)	10 (26.32%)	5 (13.16%)	0.254
Atrophy in CT	27 (71.05%)	14 (36.84%)	13 (34.21%)	0.508

Data expressed as N (%) or medians and interquartile ranges (IQR).

ADS, Alcohol Dependence Syndrome; ADS+ASPD, Alcohol Dependence Syndrome with comorbid Antisocial Personality Disorder; CT, computed tomography; N, number. Bold values are statistically significant (p<0.05).

Psychological characteristics of all subjects with a comparison between subjects with ADS and subjects with ADS+ASPD are reported in **Table 2**. Subjects with ASPD had higher scores of stress in DASS-42, traumatic events in ACE-IQ, lower scores in MoCA, more often had an addicted family member according to ACE-IQ, mental illness in the household according to ACE-IQ and incarcerated household member according to ACE-IQ (all *P*<0.05).

**Table 2:** Psychometric characteristics of participants with ADS and with ADS-ASPD.

	All (*N*=80)	ADS (*N*=52)	ADS+ASPD (*N*=28)	*P-*value
Depression in DASS-42	11 (3-30)	8.5 (0.5-24.5)	21 (6.5-25.5)	0.38
Anxiety in DASS-42	13 (5.5-24)	12 (3-21.5)	15.5 (8.5-28)	0.63
Stress in DASS-42	17 (4.5-28)	10.5 (2.5-26.5)	20.5 (12.5-35.5)	**0.026**
Traumatic events in ACE-IQ	4 (2-6)	3.5 (1-5)	6 (4.5-7)	**0.015**
MoCA score	20.5 (17-26)	23 (18-26)	19 (14.5-22.5)	**0.046**
AUDIT score	26.5 (17-34)	22 (13-31)	33 (21.5-37.5)	0.052
PSQI score	7 (3-14)	6 (3-13.5)	7 (3-16)	0.058
MMQ score	100 (74-100)	100 (82.5-100)	79.5 (55-100)	0.073
Addicted family member in ACE-IQ	46 (57.5%)	21 (26.25%)	25 (31.25%)	**<0.001**
Mental illness in the household in ACE-IQ	23 (28.75%)	9 (11.25%)	14 (17.5%)	**0.002**
Incarcerated household member in ACE-IQ	14 (17.5%)	4 (5%)	10 (12.5%)	**0.002**

Data expressed as N (%) or medians and interquartile ranges (IQR).

ADS, Alcohol Dependence Syndrome; ADS+ASPD, Alcohol Dependence Syndrome with comorbid Antisocial Personality Disorder; DASS-42, Depression, Anxiety, and Stress Scale-42; ACE-IQ, Adverse Childhood Experiences – International Questionnaire; MoCA, Montreal Cognitive Assessment; AUDIT, Alcohol Use Disorders Identification Test; PSQI, Pittsburgh Sleep Quality Index; MMQ, Multifactorial Memory Questionnaire; N, number. Bold values are statistically significant (p<0.05).

### 3.2 Bivariate analyses

The results of bivariate analyses showed that the presence of ASPD was predicted by a higher number of hospitalizations (OR = 1.13, 95% CI = 1.03-1.23, *P* = 0.007) and by a lower grade of education (OR = 0.29, 95% CI = 0.11-0.76, *P* = 0.012).

In bivariate analyses, subjects with ASPD had lower MoCA scores (OR = 0.29, 95% CI = 0.11- 0.76, *P* = 0.039), their family members were more often addicted (OR = 12.30, 95% CI = 3.29- 46.03, *P* < 0.005), had mental illness in the household (OR = 6.67, 95% CI = 1.85- 23.97, *P* = 0.004) and an incarcerated household member (OR = 4.78, 95%CI = 1.70-13.41, *P* = 0.003).

### 3.3 Multivariate analyses

Multivariate regression analyses were performed to examine whether significant results persisted after controlling for potential cofounders.

Multiple logistic regression models and results:

- In a model adjusting for the presence of ASPD (SCID-II), cognitive impairment (in MoCA), sex, length of drinking, and depression (in DASS-42), the presence of ASPD was significantly predicted by depression (OR 1.6; 95% CI: 1.14-2.23; *P* = 0.006) and cognitive impairment (OR 6.18; 95% CI: 1.9- 20.15; *P* = 0.003).
- In a model adjusting for the presence of ASPD (SCID-II), sex, length of drinking, both cognitive impairment (in MoCA) (OR 3.64; 95% CI: 1.24- 10.71; *P* = 0.019) and addicted family member (in ACE-IQ) (OR 5.76; 95% CI: 1.51- 21.99; *P* = 0.01) were independently associated with ASPD.
- In a model adjusting for the presence of ASPD (SCID-II), sex, length of drinking, both cognitive impairment (in MoCA) (OR 5.22; 95% CI: 1.62- 16.83; *P* = 0.006) and an incarcerated household member (in ACE-IQ) (OR 14.26; 95% CI: 3.49- 58.37; *P* < 0.001) were independently associated with ASPD.

## 4 Discussion

The primary finding of the present study is that 35% of study participants met the criteria for ASPD and featured distinct demographical, clinical, and psychological characteristics. Our findings are in line with previous reports suggesting that antisocial behavior is associated with an increased risk of alcohol use and predicts later problems with alcohol use (25). These findings also provide additional evidence for the high prevalence of ADS in individuals with ASPD (3). Data from a large epidemiological study of psychopathology highlights the intertwined nature of ADS and personality disorders (26). In the National Epidemiologic Survey on Alcohol and Related Conditions (NESARC), 42% of individuals who met the diagnostic criteria for any personality disorder also fulfilled the criteria for DSM-IV alcohol dependence (26). Those with ASPD showed a notably higher likelihood, being 7 to 8 times more prone to meeting the criteria for alcohol dependence (26).

An existing personality disorder elicits environmental responses, such as interpersonal or occupational problems, that provoke the onset of ADS (10). The comorbidity may result from overlapping genetic and environmental underpinnings (10). Shared environmental risk factors such as childhood trauma, coercive parenting, and antisocial peer affiliation are associated with the ADS comorbid with ASPD (10). On the other hand, ASPD incapacitates one’s ability to ignore the immediate reward offered by alcohol (10). This jeopardizes the ability to inhibit habitual use of alcohol regardless of the significant psychological and physical distress it causes, leading to the genesis of ADS (10). Conversely, excessive alcohol consumption may lead to a neuroadaptation that results in increased impulsivity or negative emotionality, as seen in ASPD (10).

On the other hand, earlier substance abuse causes aggression and/or antisocial behavior and may transform into ASPD later in life, further increasing aggression (27). Although the literature is limited in this aspect, it may be reasonable to suggest that the search for stimulus may be the initial reason that adolescents succumb to drug use, leading to antisocial/violent behavior and finally forming a vicious cycle embodied by ASPD (27). Findings indicate that proneness to act out aggressively may be linked to a reduced differentiation (at a neural and behavioral level) between threatening and non-threatening interpersonal cues, in line with the hypothesis of a hostile filter that biases the perception of the entire social environment, thus increasing the likelihood for aggressive encounters (28). Alterations of fronto-temporal-limbic regions and neuromodulatory systems, such as the serotonergic or endocannabinoid signaling systems, may also connect impulsive behavior to aggressive responding (28). Weaker cortico-striatal connectivity could relate to greater risk-taking and greater proclivity for violence (28).

**Figure 1:**
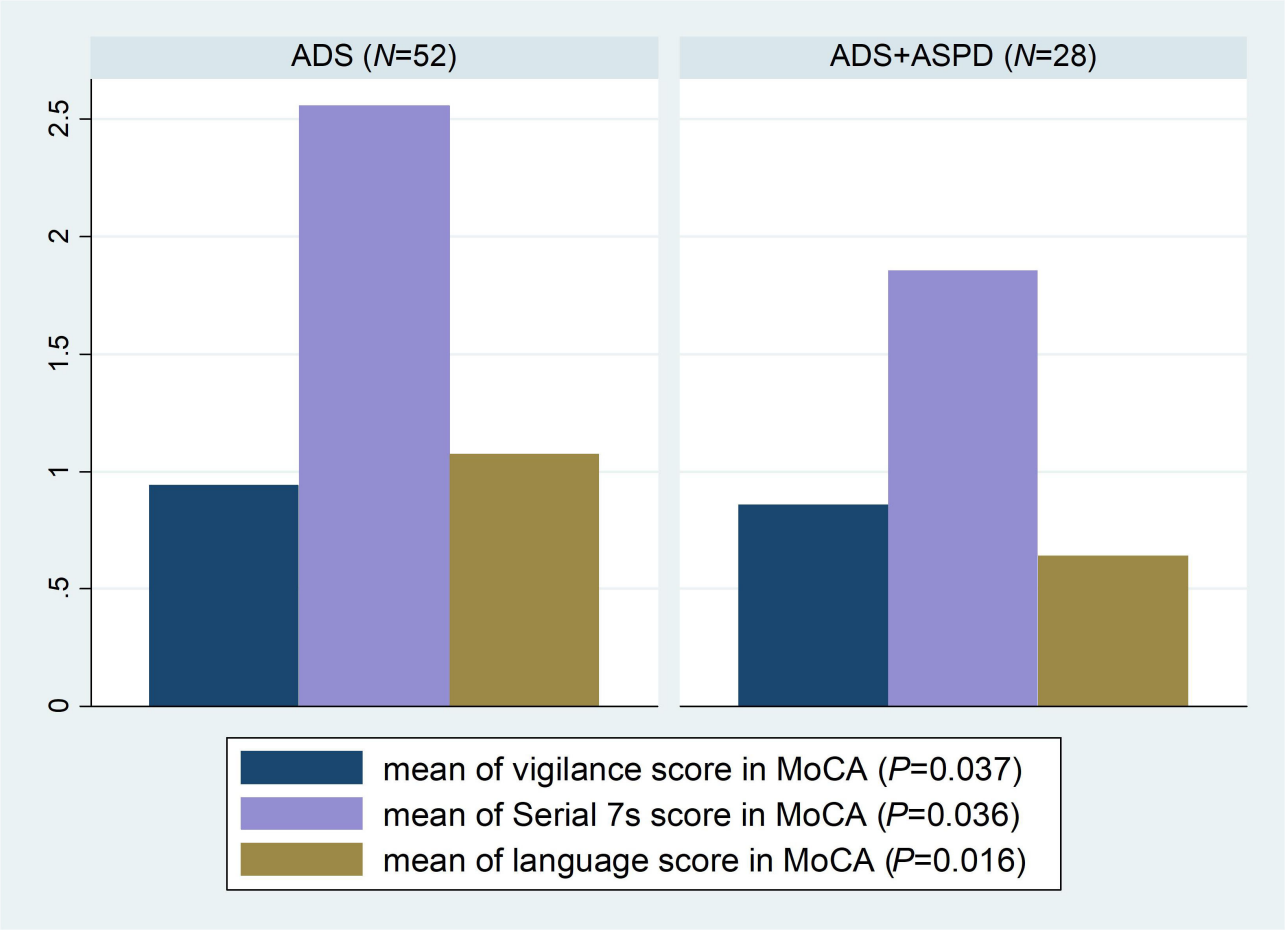
Boxplot of statistically significant MoCA subtests with a comparison between subjects with ADS and ADS+ASPD. ADS, Alcohol Dependence Syndrome; ADS+ASP, Alcohol Dependence Syndrome with comorbid Antisocial Personality Disorder; MoCA, Montreal Cognitive Assessment; N, number.

Furthermore, we found that participants with ASPD had lower MoCA scores and had more problems with vigilance, attention, and linguistic skills (**Figure 1**). This is in line with previous literature findings that antisocial symptoms have been significantly associated with cognitive control defects, attentional problems, abnormalities in decision-making, deficits in flexible responding, such as reversal learning, planning impairments, neural regions governing inhibitory control, and verbal deficits/delayed language development (29, 30). The young offenders scored approximately half a standard deviation lower on verbal domains of intelligence than on performance-related domains of general intelligence (31). Moreover, in subjects with ASPD, spatial convergence in brain regions belonging to ventral and dorsal attention networks (the anterior midcingulate cortex/the pre-supplementary motor area (aMCC/pre-SMA), superior parietal lobule (SPL), and premotor (frontal eye field, and the cuneus) were found (32). Indeed, the aMCC/pre-SMA mainly corresponded to the ventral network, whereas the SPL and premotor cortex were associated with the dorsal network (32). These regions are frequently co-activated in tasks requiring attentional processes such as shifting: sustained, oddball, and working memory (32). Ventral network regions (e.g., aMCC/pre-SMA) may correspond to a stimulus-driven attentional system. In contrast, regions subserving the dorsal attentional network (e.g., SPL, premotor cortex) may play a crucial role in top-down or goal-driven attentional control (32). Subjects with ASPD have significant connectivity deficits in the amygdala, middle cingulate cortex, ventral posterior cingulate cortex-precuneus, ventromedial and dorsomedial prefrontal cortex, premotor cortex, and superior parietal lobule and increased connectivity in the ventral posterior cingulate cortex and decreased connectivity in the parietal operculum, calcarine cortex, and cuneus (32). There is a negative relationship between the severity of antisocial behaviors and connectivity with the ventromedial prefrontal cortex (32). These findings overlapped with socio-affective and attentional processes (32). The ASPD is associated with variability in neural functioning during reward and loss processing (33). In particular, impulsive-antisocial traits appeared to be specifically associated with hypersensitivity in the ventral striatum (VS) and prefrontal cortex (PFC) (VS reactivity or VS-PFC functional connectivity) during the anticipation, but not the receipt of rewards (33). Beyond VS reactivity, ASPD was related to the greater orbitofrontal cortex (OFC), medial PFC reactivity, and VS-dorsomedial PFC connectivity during reward processing (33). The ASPD was linked to greater reactivity in several regions, including the posterior cingulate, precuneus, and insula, during error-related loss receipt and decreased VS reactivity during loss anticipation (33).

Our finding of younger age among ADS participants with comorbid ASPD corresponds with the literature (34). In our sample, individuals diagnosed with ASPD tend to start drinking alcohol at younger ages, which is in line with literature findings (35), and were more likely to seek treatment at a younger age, which was previously described (10). Studies also reported that prevalence rates of ASPD steadily decline with age (36). Over the years, researchers have proposed multiple factors that may contribute to the decline in the prevalence of ASPD with age, including increased rates of mortality and incarceration and a change in personality traits over the lifespan (37).

We found that individuals with ASPD had more frequent hospitalizations and more problems with alcohol consumption, as shown in the AUDIT test, which aligns with literature findings - more than half of subjects dependent on alcohol relapse within a year (38). Relapse can be attributed to several psychosocial and biological factors. Among others, marital status, traumatic experiences, psychological distress, and the presence of co-existing psychopathology, including anxiety and depression, and alteration in the brain’s dopamine system may influence relapse (10). If individuals with ASPD seek treatment, their chances of adhering to the treatment are low because they tend to violate rules (10). The comorbidity leads to poorer treatment outcomes, thereby increasing the chance of relapse or drop-out (10).

We reported that subjects with comorbid ASPD had lower education levels, tended to be unemployed more often, and tended to be married less often. The results align with the literature finding, which revealed significantly higher odds of a diagnosis among individuals with less than a high school education, lower income, and those not married (37). Using the NESARC-III data, Goldstein et al. (5) found similar results with adults of any age. In their study, individuals with ASPD were more likely to be 18-29 years old, widowed/separated/divorced, had less than a college degree and a lower family income (5). Also, more knowledge on the effect of education level on this relation is required since research suggests that school failure is overrepresented in offenders and related to aggressive antisocial behaviors (39). It is hypothesized that behavioral disorders and impulsivity in individuals with ASPD negatively affect their academic and professional achievements, which results in lower income levels and employment compared to healthy individuals (40).

We reported that participants with ADS+ASPD more often have family members who suffer from ADS, are incarcerated, or are otherwise mentally ill. Similarly, a state hospital study (41) of 740 individuals admitted for alcoholism treatment concluded that ADS+ASPD subjects had a higher prevalence of familial alcoholism when compared with alcohol abusers without ASPD. This points us toward the etiopathogenesis of the disease, which combines genetic and environmental influences (42–44). Notably, Rhee and Waldman’s (45) meta-analysis reported additive and non-additive genetic contributions to antisocial behavior (32% and 9%, respectively), 16% of the variance explained by shared environment, and 43% explained by individual-specific environmental influences. A genetic basis for ASPD is supported by the finding that a first-degree biological relative with ASPD constitutes a risk factor for the disorder (43). The ASPD, like most mental disorders, is thought to have a complex, multifactorial etiology characterized by polygenic inheritance and genetic heterogeneity across individuals. The genetic contribution is not stable over time, suggesting that partly different genes contribute to ASPD at different stages of the lifespan (44). Recently, GWAS meta-analysis identified a novel genome-wide significant signal with rs9806493 chromosome 15q26.1 close to SLCO3A1, possibly contributing to the genetic risk factor for ADS and ASPD (42). Many environmental risk factors were described in the literature. Of the prenatal risk factors, maternal smoking, alcohol use, drug use, and stress during pregnancy are among the best documented (44). Perinatal risk factors include obstetric complications, parental psychopathology, malnutrition, and exposure to heavy metals (44). Postnatal risk factors include more strained family relationships, child maltreatment, parental substance abuse, lower income, childhood residential mobility, and bullying (43).

**Figure 2:**
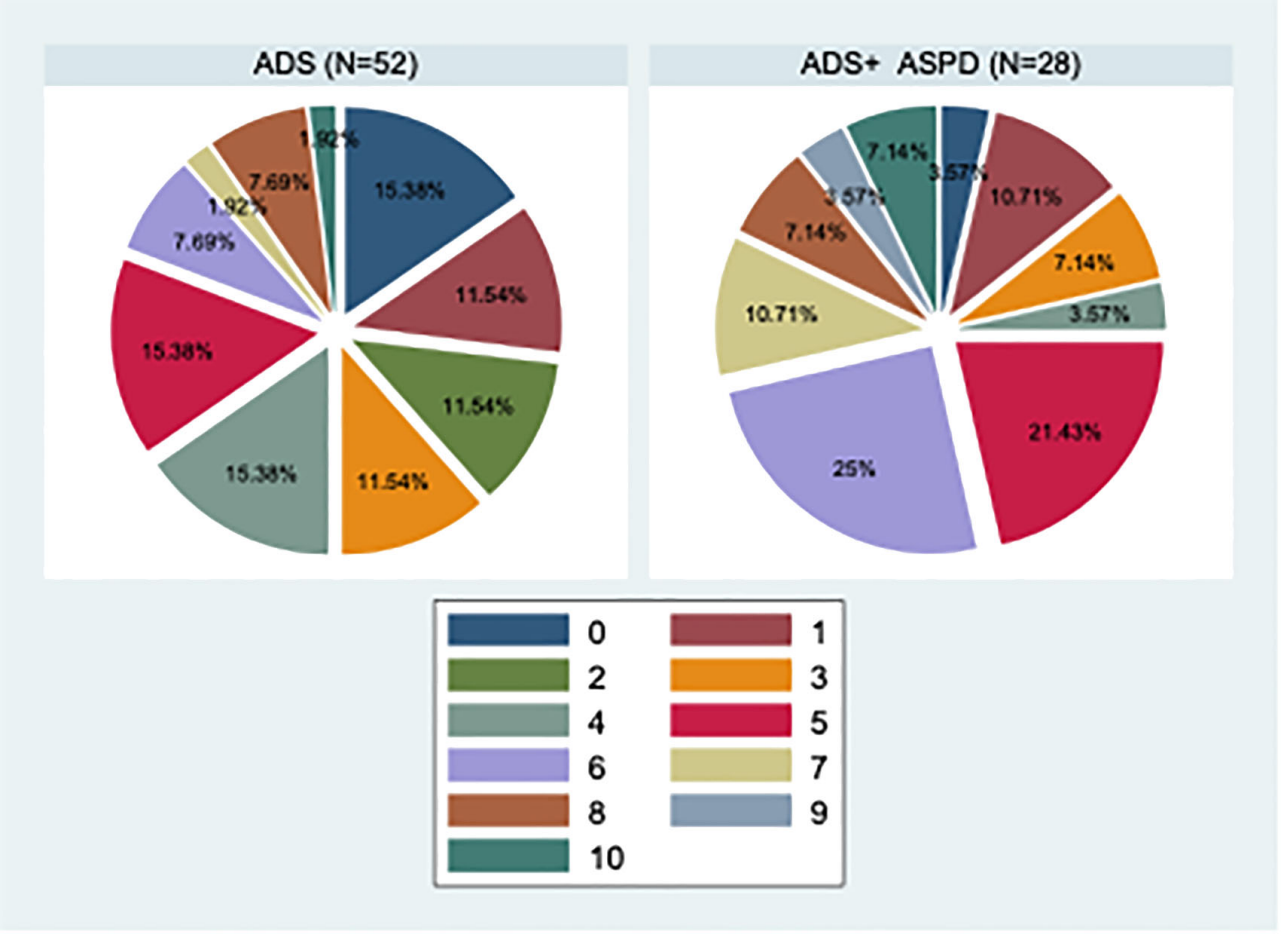
Pie graphs of the number of different types of traumatization in ACE-IQ with a comparison between subjects with ADS and ADS+ASPD. ADS, Alcohol Dependence Syndrome; ADS+ASPD, Alcohol Dependence Syndrome with comorbid Antisocial Personality Disorder; N, number.

Subjects with ASPD reported experiencing traumatic events more often (**Figure 2**), which is in agreement with previous literature findings (10). Adverse childhood experiences, which encompass various forms of abuse, neglect, and impoverishment during childhood, are prime examples of negative socialization experiences that engender antisocial traits and behaviors (2). Clinical evidence suggests that trauma may contribute to ASPD features such that the experience of trauma provides a template for which people who develop ASPD learn to interact with others (46). People with ASPD traits may use the betrayal trauma they experienced earlier in life as a template for perpetrating trauma on others later in life (2). One way of understanding the relationship between antisocial behaviors preceded by traumatic experiences is to suggest that individuals who have experienced severe trauma develop insensitivity to stressful situations. In addition to numbing reactions of sadness and fear, these individuals would tend to behave aggressively. Traumatic experiences compromise the functioning of neurotransmitters, the neuroendocrine, and the immune system, generating a dysfunction of the hypothalamic-pituitary-adrenal system affecting control and stress response (4). The average correlation between trauma and ASPD traits were both high. Exposure to traumas at all levels of betrayal predicts ASPD traits for women, but only exposure to high betrayal trauma predicts ASPD traits for men (2). Fergusson et al. (47) examined the association between childhood sexual abuse, childhood physical abuse, and ASPD and found that the prevalence of ASPD at ages 18–21 and 21–25 was two to four times greater among those who had been sexually abused compared to those who had not. Similarly, those who experienced regular physical abuse or severe physical abuse had ASPD at a prevalence that was two to seven times higher than those who were not physically abused. In multivariate models, sexual abuse predicted ASPD; however, physical abuse did not occur in the fully adjusted model (2).

**Figure 3:**
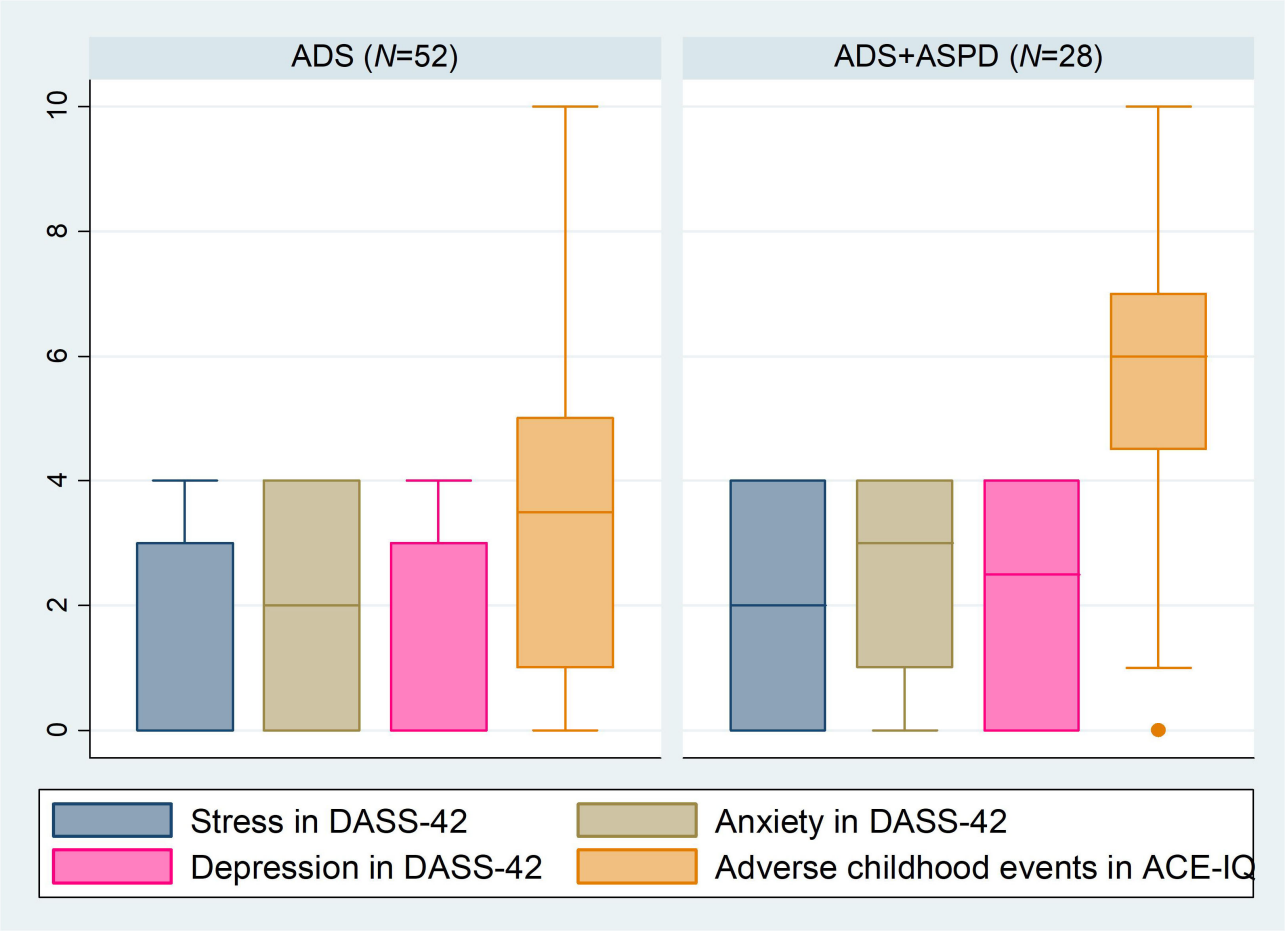
Boxplot of selected questionnaire results with a comparison between subjects with ADS and ADS+ASPD. ADS, Alcohol Dependence Syndrome; ADS+ASPD, Alcohol Dependence Syndrome with comorbid Antisocial Personality Disorder; DASS-42, Depression, Anxiety, and Stress Scale-42; ACE-IQ, Adverse Childhood Experiences - International Questionnaire; N, number.

Our next finding was that depression, anxiety, and stress were all statistically significantly correlated with ASPD) (**Figure 3**), which is in line with the literature findings (40). However, we failed to replicate this finding in a linear regression model, possibly due to the small sample size. Symptoms reflecting impulsivity and emotional dysregulation are found in ASPD, psychopathy, anxiety, and mood disorders. Interestingly, the relationship between psychopathy and ASPD with mood and anxiety disorders has been a controversial topic, as many experts associate ASPD and psychopathy with very low levels of anxiety and depression (3). In contrast, in the DSM-5, it is noted that those with an ASPD diagnosis may also experience anxiety disorders and/or depressed mood (1). Evidence from epidemiological samples indicates that individuals with ASPD are four times more likely to experience a mood disorder, and up to half of the individuals with ASPD may also experience an anxiety disorder in their lifetime, particularly posttraumatic stress disorder and social anxiety disorder. These individuals with comorbid ASPD and anxiety disorders were found to be at increased risk for major depression and substance dependence (48, 49). Drinking is sometimes used as a coping strategy and/or method of self-medication among individuals with substance abuse disorder before, during, and after anxiety-provoking events (3).

Our last finding was that individuals with ADS-ASPD reported worse sleep, which showed a statistical trend. Previous sleep problems were reported in individuals with ASPD and ADS (50). A similar relationship between sleep and ASPD was described in a study conducted by Van Veen in 112 patients with antisocial personality disorder and borderline personality disorder. The study found a relationship between behavioral disinhibition and/or emotional dysregulation and poor sleep. Poor sleep quality was reported in 53.6% of the patients, especially with problems falling asleep, and 22.3% suffered from severe chronic insomnia, which was particularly characterized by major problems with sleep initiation, resulting in much shorter total sleep times. Problems with sleep were significantly associated with greater impulsivity. Subjects with greater insomnia or lower sleep quality reported more difficulties with focusing and controlling their thoughts (51). However, to our knowledge, this is the first study comparing sleep problems in individuals with ADS with and without ASPD.

A recent meta-analysis on 24,801 subjects did not find enough evidence to determine whether or not medication is a helpful treatment for people with ASPD (52). There is also minimal evidence available on psychological interventions for adults with ASPD (53). A recent meta-analysis of 605 adults found that three interventions (schema therapy, contingency management, and dialectic behavior therapy) may be more effective than other approaches (53). However, the certainty of the evidence was low or very low to recommend or reject any psychological treatment for people with a diagnosis of ASPD (53).

Our findings may not apply to all participants with alcohol dependence syndrome as the sample we studied was small. Nevertheless, the group was homogenous; all subjects were from the same region, and the same team of psychiatrists evaluated them. The results might differ in a sample of outpatients with the same problems and a sample of people recovering from ADS. Many of the participants in the present study were using benzodiazepines at the time of the examination, which can worsen some cognitive parameters.

Since this study extensively uses questionnaire methods, it was not possible to objectively evaluate variables such as sleep parameters or the incidence of traumatization. Specifically, in cases of memory decline, they may be unable to recall all the events. In addition, a limited insight into one’s problems is very common in this population. The present results are preliminary, and the study is still ongoing. Further studies are warranted to evaluate the impact of various comorbidities and their treatment on cognitive skills.

The strength of the current study is that it provides a comprehensive analysis of comorbid ASPD among adults with ADS based on the in-depth analysis of the selected demographic and psychological variables controlling for key covariates in analyses. Another strength is that it explored familial psychopathology among subjects, providing deeper insights into their conditions. Overall, the study advances understanding of the complexities of comorbid ASPD in individuals with ADS, providing valuable insights for research and clinical practice.

The study demonstrated that more than one-third of the individuals with ADS met the criteria for ASPD. The prevalence of depression, anxiety, stress, traumatization, and sleep problems was high among all subjects. All these difficulties were closely related to ASPD. Participants with ADS+ASPD had lower MoCA scores and had more problems with attention and linguistic skills. The findings implicate that screening for ASPD might be helpful in the complex management of ADS.

## Data Availability

The raw data supporting the conclusions of this article will be made available by the authors, without undue reservation.
